# Peritoneal dialysis in lupus nephritis: comparative outcomes vs. hemodialysis, risk stratification, and modality selection framework

**DOI:** 10.1080/0886022X.2026.2670917

**Published:** 2026-05-20

**Authors:** Shuiqin Cheng, Dandan Peng, Xia Wang, Le Yu, Guisheng Ren, Zhihong Zhang, Zhengzhao Liu

**Affiliations:** National Clinical Research Center of Kidney Diseases, Jinling Hospital, Affiliated Hospital of Medical School, Nanjing University, Nanjing, China

**Keywords:** Lupus nephritis, peritoneal dialysis, hemodialysis, end-stage kidney disease, residual renal function, cardiovascular outcomes

## Abstract

Peritoneal dialysis (PD) is an effective and safe renal replacement therapy (RRT) for lupus nephritis (LN) patients with acute kidney injury (AKI) or end-stage renal disease (ESRD). This narrative review compares PD and hemodialysis (HD) in LN across both AKI and ESRD settings, highlighting differences in survival, cardiovascular outcomes, infection risk, and lupus flare incidence. We summarize clinical evidence demonstrating the preservation of residual renal function (RRF), hemodynamic stability, and improved cardiovascular tolerance associated with PD, while also noting challenges such as higher peritonitis susceptibility in chronically immunosuppressed patients. A structured risk stratification framework is presented to aid selection between PD and HD based on cardiac comorbidities, RRF, infection risk, catabolic state, pediatric status, and ability to perform home-based therapy. In addition, we outline a clinical decision algorithm to guide modality selection and discuss immunosuppressive strategies in PD-treated LN patients. Despite comparable mortality between PD and HD in most studies, PD may offer superior cardiovascular protection and lower flare risk in selected LN phenotypes. Early identification of ideal PD candidates, individualized prescription strategies, and multidisciplinary management are crucial to optimizing outcomes. This review provides a practical comparative synthesis, a decision-making framework, and future perspectives to support clinicians managing LN patients requiring dialysis.

## GRAPHICAL ABSTRACT

**Figure:**
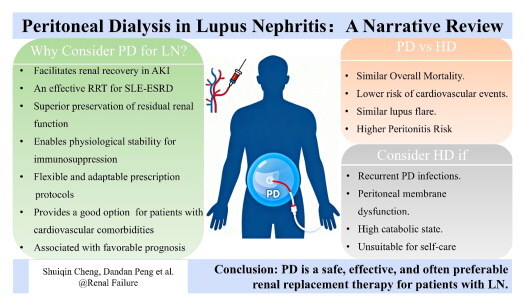

## Introduction

Lupus nephritis (LN) is a common complication of systemic lupus erythematosus (SLE), which can lead to varying degrees of renal impairment. Proteinuria, younger age, low C3, among others, are strong predictors of renal failure at the time of SLE diagnosis [1]. Achieving sustained clinical remission and maintaining it for an extended duration are protective against the decline in kidney function [2]. The incidence of kidney replacement therapy initiation for kidney failure among SLE patients was 0.80 per million population per year, with the incidence remaining stable [3]. Approximately, 5–30% of LN patients progress to end-stage renal disease (ESRD) within 10 years after diagnosis [4], necessitating renal replacement therapy (RRT) options like hemodialysis (HD), peritoneal dialysis (PD), or kidney transplantation (KT) [5,6]. Patients with LN who progress to ESRD or require dialysis incur higher medical costs [7,8]. For those patients who have developed ESRD due to LN, a classic form of immune-mediated ESRD, PD serves as an effective method of RRT. In recent years, the clinical application of PD in the treatment of LN has attracted increasing attention, particularly regarding its potential for PD-driven immunomodulation.

However, due to the specificity of the primary disease, LN patients undergoing dialysis often have lower serum albumin levels, are more prone to infections, and experience a poorer quality of life. Moreover, these patients are often treated with steroids [9], which makes them more susceptible to complications during the clinical treatment process, affecting the outcome of the disease. Siu et al. [10] showed that patients with SLE on continuous ambulatory peritoneal dialysis (CAPD) exhibit significantly lower pre-dialysis serum albumin levels and require higher doses of erythropoietin to achieve comparable hemoglobin levels compared to other non-diabetic chronic glomerulonephritis CAPD patients. Additionally, they face a poorer prognosis regarding infectious complications and mortality rates, indicating that LN patients undergoing PD treatment still face many challenges.

Clinically, the application of PD in the treatment of LN requires a comprehensive consideration of the patient’s specific situation. The key to treatment is the formulation of personalized plans, including the choice of PD modality, the type of dialysate, and the management of related complications. Additionally, immunosuppressive treatment for LN patients is a critical component of the treatment plan to reduce autoimmune responses and slow down further deterioration of kidney function, managing LN itself involves immunosuppressive therapies, which can complicate the patient’s overall care plan. Coordination between nephrologists, rheumatologists, and other specialists is crucial to optimizing treatment outcomes for these patients. This review aims to review the advantages and challenges of PD in the treatment of LN, compare the clinical outcomes of PD and HD, and discuss the use of immunosuppressants and strategies for optimizing PD prescription.

## Methods

This narrative review was conducted based on a systematic literature search of PubMed, Embase, and the Cochrane Library for articles published between January 2000 and January 2025. The search strategy utilized keywords and MeSH terms related to ‘peritoneal dialysis,’ ‘lupus nephritis,’ ‘outcomes,’ and ‘immunosuppression.’ After duplicate removal, 120 records were initially identified. Through title/abstract screening and subsequent full-text review, 43 studies were deemed eligible and included in this synthesis. The inclusion criteria focused on original studies reporting on LN patients treated with PD. Non-English articles and studies not focusing on LN outcomes were excluded. Data on study design, patient characteristics, and clinical outcomes were extracted and critically evaluated.

## PD in LN patients with AKI

The application of PD in treating LN with concurrent acute kidney injury (AKI) provides a new treatment option for this specific patient group. LN, a severe complication of SLE, can lead to rapid renal failure in patients. When this nephritis progresses to concurrent AKI, the difficulty and complexity of treatment significantly increase, necessitating prompt and effective interventions to prevent further deterioration of renal function. As a gentle and continuous form of RRT, PD compared to HD, has a lesser impact on a patient’s circulatory stability and is particularly suitable for patients with AKI.

For patients with LN and concurrent AKI, PD not only effectively removes toxins and excess fluid from the blood but also reduces the burden on the kidneys, creating favorable conditions for renal recovery. In pediatric lupus nephritis-acute kidney injury (LN-AKI), Stotter et al. [11] found that 79.7% of children initially received intermittent HD for fluid overload, but transitioned to PD during the recovery phase, with 82% achieving partial renal function recovery.

Furthermore, PD provides gradual and continuous metabolic waste and fluid clearance, helping to maintain electrolyte and acid–base balance, which is especially important for patients with LN and concurrent AKI. PD allows for a more flexible treatment plan, enabling adjustments to the frequency and duration of dialysis based on the specific circumstances and needs of the patients. Zhou et al. [12] showed that PD can be an effective treatment for patients with severe LN associated with AKI and accompanied by critical organ dysfunction. PD not only alleviates symptoms such as edema and heart failure but also helps preserve residual renal function (RRF) and enhances the patients’ nutritional status.

Additionally, since PD can be performed at home, it reduces the burden on patients related to traveling to and from the hospital, thereby improving their quality of life. However, patients with LN and concurrent AKI who are treated with PD also face challenges, including the risk of PD-related infections and issues with patient tolerance.

## PD in LN patients with CKD/ESRD

Chronic kidney disease (CKD) is one of the most severe complications in patients with LN, and progression to ESRD indicates a poor prognosis. The economic implications of ESRD extend far beyond direct medical expenses; they also encompass significant indirect costs to society, such as the loss of productivity experienced by both patients and their caregivers [13]. Renal treatment is a set of coordinated, multifaceted interventions aimed at optimizing a renal patient’s physical, psychological, and social well-being, while also working to stabilize, slow, or even reverse the progression of renal deterioration – ultimately reducing ESRD morbidity and mortality [14]. Once ESRD is reached, a dialysis option needs to be chosen. Choosing the appropriate dialysis method (HD or PD) is particularly important.

Advantages of PD in LN with CKD: (1) preservation of residual kidney function: RRF preservation is a key PD strength; PD is often associated with a slower decline in residual kidney function compared to HD. This is particularly beneficial for patients with LN, as preserving kidney function can improve outcomes and delay the need for more intensive treatments. Chen et al. [15] showed PD patients maintained RRF (urine output >200 mL/d) for a median of 14 months vs. 6 months in HD patients. (2) *Flexibility and autonomy*: PD allows patients more flexibility and autonomy in their treatment schedules compared to the more regimented schedules required by in-center HD. This can significantly improve the quality of life for patients with LN and CKD, who often deal with various other medical appointments and treatments [11]. (3) *Reduced hemodynamic stress*: Because PD occurs more gradually over a longer period, it places less hemodynamic stress on the cardiovascular system than HD. This is especially important for LN patients, who may already have an increased risk of cardiovascular disease (CVD). (4) *Immunological considerations*: The immunological aspects of LN mean that minimizing exposure to potential external immunological triggers is beneficial. PD performed at home, can reduce the risk of infections associated with in-center treatments and exposure to hospital or clinic environments. *Challenges in application*: Despite these advantages, the application of PD in LN patients with CKD is not without challenges. These include the risk of PD-related infections, potential complications from long-term PD, and the need for rigorous training and adherence to treatment protocols by the patient and their caregivers.

Gonzalez-Pulido et al. [16] demonstrated that when a patient with LN progresses to ESRD, SLE activity often enters a phase of quiescence or ‘burn-out,’ particularly when the patient begins receiving RRT. Ideally, this reduction in disease activity is facilitated by the administration of immunosuppressive therapy, as illustrated by Maroz and Segal [17].

PD provides a viable and effective RRT option for patients with LN and concurrent CKD. It offers several advantages that can improve patient outcomes and quality of life. However, careful patient selection, comprehensive education, and close monitoring for potential complications are essential to maximize the benefits of PD in this patient population.

In systemic lupus erythematosus-end-stage renal disease (SLE-ESRD), PD serves as an effective RRT option, though long-term outcomes require careful evaluations: mortality and cardiovascular outcomes, technique survival and transplantation bridge. These evaluations are based on comparisons with HD.

## Comparison with HD

**Figure 1.**
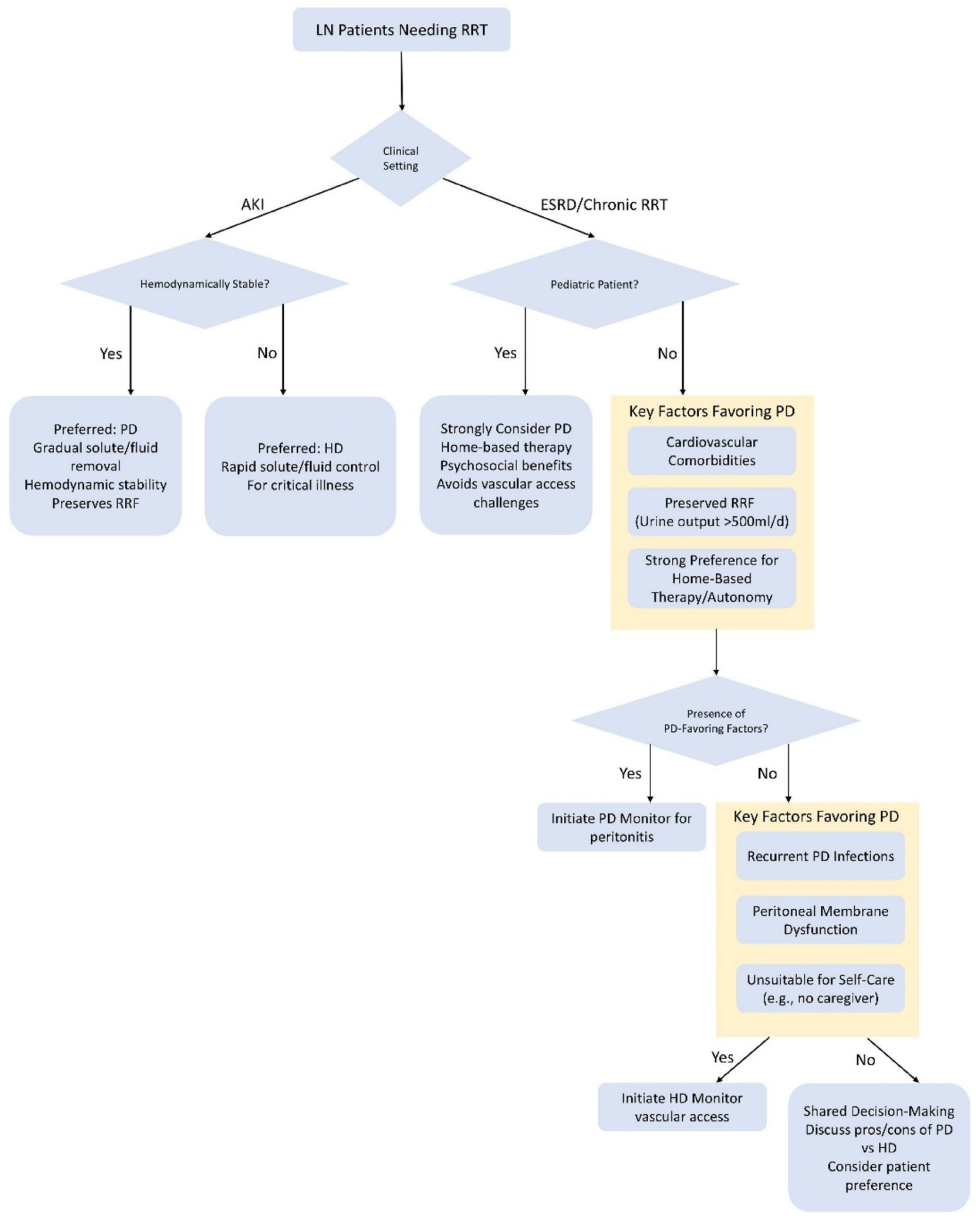
Clinical decision pathway for selecting PD vs. HD in Ln with AKI or ESRD. Clinical decision pathway for selecting PD vs. HD in LN patients requiring RRT. This algorithm guides modality selection based on the clinical presentation (AKI vs. ESRD), patient-specific factors, and key contraindications. For AKI, hemodynamic stability is the primary determinant, favoring PD for stable patients to preserve RRF and ensure hemodynamic stability. For ESRD, pediatric status strongly favors PD. In non-pediatric adults, the decision incorporates a balance of factors favoring PD (e.g., cardiovascular comorbidities, preserved RRF, and preference for home therapy) vs. those favoring HD (e.g., recurrent PD infections, peritoneal membrane dysfunction, and lack of self-care capability). The pathway culminates in shared decision-making when no strong factors are present, emphasizing patient preference and a detailed discussion of the pros and cons of each modality. LN: lupus nephritis; RRF: residual renal function; AKI: acute kidney injury; ESRD: end-stage renal disease; PD: peritoneal dialysis; AKI: acute kidney injury; HD: hemodialysis.

Given the elevated risk of vascular complications in patients with SLE, it is not surprising that this retrospective cohort study [18] revealed a significantly higher long-term incidence of arteriovenous fistula/graft (AVF/AVG) dysfunction in this population. In contrast, PD eliminates the need for vascular access and its associated risks. To enable a more comprehensive comparison between PD and HD, we have summarized key outcomes in Table 1, which provides a comparative analysis of PD and HD in patients with LN, including mortality, cardiovascular event risk, infectious episodes, and lupus flare. The reasons for potential heterogeneity among different studies include baseline characteristics of study populations, vascular access management, comorbidities, regional healthcare quality, statistical models, and other factors. Therefore, the interpretation of comparative outcomes between PD and HD should be contextualized within these potential sources of variation. And we have summarized clinical decision pathway for selecting PD vs. HD in LN with AKI or ESRD in Figure 1.

**Table 1.** Comparative analysis of PD vs. HD in LN patients.

Study	Year	Design	Country	Sample size (PD vs. HD, *n*)	Key outcomes assessed PD outcome vs. HD	Conclusions	Limitations
Daoud et al. [19]	2025	Meta-analysis	Global	SLE-ESRD (total 307)	Similar (lupus flares)	No significant difference in lupus flare risk between PD and HD.	No specific patient numbers for PD and HD: potential heterogeneity in flare definition.
Lao et al. [20]	2024	Retrospective study	Australia and New Zealand	SLE-ESRD (68 vs. 137)	PD (lower mortality)	PD associated with lower all-cause mortality compared to HD in this cohort.	Observational design; potential for residual confounding
Gou and Tuo [21]	2022	Meta-analysis	Global	SLE-ESRD (564 vs. 2,841)	Similar (mortality)	No significant difference in all-cause mortality between PD and HD.	Significant heterogeneity among included studies; limited data on cause-specific mortality.
Kim et al. [22]	2022	Retrospective study	Korea	SLE-ESRD (25 vs. 96)	Similar (flare and mortality)	Comparable rates of disease flare and mortality between PD and HD.	Small sample size in PD group; single-center design.
Swai et al. [23]	2020	Meta-analysis	Global	SLE-ESRD (2,089 vs. 4,616)	PD (lower risk of cardiovascular events)	PD associated with a lower risk of cardiovascular events compared to HD.	Unable to adjust for all potential confounders across studies; heterogeneity in cardiovascular outcome definitions.
Tsai et al. [24]	2019	Retrospective study	Taiwan	SLE-ESRD (12 vs. 42)	PD (lower lupus flare)	PD associated with a specifically lower risk of lupus flare compared to HD.	Very small sample size; single-center; retrospective design with potential selection bias.
Contreras et al. [25]	2014	Retrospective study	American	SLE-ESRD (1,355 vs. 9,668)	Similar (mortality)	No significant difference in mortality between PD and HD after extensive adjustment.	Observational design; potential for unmeasured confounding despite propensity score matching.
Chang et al. [26]	2013	Retrospective study	Taiwan	SLE-ESRD (260 vs. 813)	PD (mortality, female HD equivalence, male HD disadvantage)	PD associated with better survival in male LN patients; HD associated with worse survival in males but not females.	Findings may not be generalizable to non-Asian populations; unmeasured confounders related to vascular access management.
Kang et al. [27]	2011	Retrospective study	Korea	SLE-ESRD (14 vs. 28)	Similar (total prognosis)	No significant difference in overall patient survival between PD and HD, though PD had lower technique survival.	Small sample size; single-center; short follow-up period.
Weng et al. [28]	2009	Retrospective study	Taiwan	SLE-ESRD (22 vs.14)	HD (lower mortality and numbers of infectious episode)	HD associated with lower mortality and fewer infectious episodes compared to PD.	Very small sample size; high risk of bias due to nonrandom allocation and incomplete adjustment for disease severity

PD: peritoneal dialysis; HD: hemodialysis; LN: lupus nephritis; SLE-ESRD: systemic lupus erythematosus-end-stage renal disease.

The majority of studies demonstrate no significant difference in all-cause mortality between SLE-ESRD patients undergoing PD and those on HD. A meta-analysis by Gou and Tuo [21] (*n* = 3,405) demonstrated no significant difference in all-cause mortality between PD and HD, with comparable rates of cardiovascular and infection-related deaths. This is supported by a large registry study [25] showing similar 5-year mortality after propensity score matching (38.2% in PD vs. 39.5% in HD, *p* = 0.67). However, subgroup analysis revealed Asian LN patients on PD had a nonsignificant trend toward lower mortality, while male PD patients exhibited a 21% lower risk. Contradictory results arose in smaller studies, such as Weng et al. [28], which reported higher mortality in PD vs. HD (HR: 1.82, 95% CI: 1.03–3.21). This study’s high risk of bias stemmed from nonrandom patient allocation and incomplete adjustment for disease severity (Newcastle-Ottawa Scale score: 4/9). A Taiwanese nationwide study [26] found HD was associated with worse survival in male LN patients (HR: 1.45, 95% CI: 1.12–1.88) but not in females, potentially reflecting regional disparities in vascular access management or comorbidity control. The vast majority of studies have reached consistent conclusions regarding mortality rates, with only a few exceptions. These discrepancies may be attributed to differences in study populations, regional variations in medical standards, and statistical modeling approaches.

In SLE patients with ESRD, when PD outcomes are comparable to or superior to those of HD, PD should be prioritized as the preferred option for initiating RRT. A meta-analysis [23] showed that both PD and HD provide similar clinical dialysis effects and serve as effective initial choices for RRT in LN patients with ESRD prior to renal transplant and that PD was superior to HD in terms of all-cause cardiovascular events, offering better outcomes. Most studies indicate no significant difference in mortality rates between PD and HD, though some studies suggest that PD may offer advantages in terms of cardiovascular events. Gong et al. demonstrated [5] that PD was superior to HD in terms of all-cause cardiovascular events, offering better outcomes. A systematic review and meta-analysis [26] of eight studies showed PD was associated with a 27% lower risk of all-cause cardiovascular events, attributed to its gradual fluid removal. Kang et al. [27] confirmed this, reporting that while PD had lower 5-year (70% vs. 86%) and 10-year (23% vs. 86%) technique survival than HD, PD patients had a 34% lower risk of myocardial infarction. Jorge et al. [29] found that, for lupus nephritis-end-stage renal disease (LN-ESRD) patients, dialysis followed by renal transplantation is associated with a substantially reduced risk of cardiovascular events, including myocardial infarction and cerebrovascular accident.

LN patients on PD face higher peritonitis risk than other renal disease populations, likely due to chronic immunosuppression. Huang et al. [9] reported a peritonitis rate of 0.58 episodes/100 patient-months in lupus nephritis peritoneal dialysis (LN-PD) patients, double that of non-LN PD populations (0.29 episodes/100 patient-months, *p* < 0.01). Steroid use further impairs peritoneal immune function [10].

Both of the two dialysis modalities require attention to lupus flare risk. Kim et al. [22] observed comparable flare incidences (25.3% in PD vs. 23.7% in HD), noting that hematologic manifestations such as thrombocytopenia accounted for 42% of flares. This finding is corroborated by a systematic review [23], which found no significant difference in flare risk. The prevailing consensus, therefore, is that dialysis modality does not significantly affect autoimmune activity. Despite this consensus, some studies have reported divergent findings. For instance, Tsai et al. [24], in a small-sample study, demonstrated a specifically lower risk of lupus flare in patients undergoing PD compared to HD.

In pediatric LN-ESRD, PD is the dominant initial modality. This preference is primarily due to the significant challenges associated with creating and maintaining viable vascular access in children, as well as the profound benefits of home-based therapy, which minimizes disruption to school attendance, family life, and overall psychosocial development. Wasik et al. [30] found PD was used in 68% of pediatric LN patients, with HD associated with higher 1-year hospitalization rates (adjusted OR: 2.1, 95% CI: 1.1–4.2). Stotter et al. [11] reported intermittent HD was used for acute fluid overload in 79.7% of pediatric LN-AKI cases, but 61% transitioned to PD during recovery, with 82% achieving partial renal function recovery. Notably, pediatric studies often have small sample sizes (median *n* = 87) and retrospective designs, carrying moderate risk of selection bias (e.g., HD may be reserved for more critically ill children). Prospective pediatric cohorts are needed to validate these findings. Furthermore, particular caution is warranted for adolescent patients transitioning from pediatric to adult renal care, as they face a notably higher risk of technique failure, often attributable to psychosocial challenges and non-adherence to the complex regimen [31]. Managing pediatric LN patients on PD requires a developmentally tailored approach. *Clinical pathway*: PD, particularly continuous cycling PD (CCPD), is the preferred initial modality to minimize disruption to school and daily life, with structured transition planning for adolescents. *Unique challenges*: Children face significant psychosocial stressors, including body image issues, caregiver dependency, and peer isolation. Medication non-adherence during adolescence is a leading cause of technique failure. *Neurocognitive and growth impacts*: Long-term PD may impair neurodevelopment via uremic neuroinflammation, sleep disruption from cycling, and recurrent peritonitis. Growth retardation, driven by dialysate protein loss, chronic inflammation, and malnutrition, requires intensive nutritional intervention and growth hormone therapy when appropriate. A comprehensive pediatric LN-PD program must therefore merge medical management with psychological, educational, and transition support to holistically address the interplay of health and developmental needs.

*Risk stratification*: Who benefits most from PD vs. HD? A structured risk stratification framework can guide dialysis modality selection based on clinical features (Table 2). This stratification aims to guide personalized modality selection by matching patient-specific factors with the distinct advantages of each dialysis therapy. The overarching objective of this framework is to facilitate the timely transition of stable LN-ESRD patients to KT. PD is central to this goal, as it is strongly preferred for its superior ability to preserve RRF and avoid the need for vascular access, thereby optimally bridging patients to transplant. And we summarized ‘Clinical Decision Impact Summary’ box.

**Table 2.** Risk stratification framework for selecting PD vs. HD in LN-ESRD.

Patient characteristic/clinical scenario	Rationale/key considerations	Clinical pearls
*Factors favoring PD*		
Cardiovascular comorbidities (e.g., heart failure, low ejection fraction, recent myocardial infarction) [23]	Gradual fluid removal minimizes hemodynamic stress.	Avoid HD in patients with unstable angina within 3 months.
Preserved residual renal function (urine output >500 mL/d) [15]	Associated with slower decline of RRF and improved fluid/nutritional status.	Monitor urine output monthly to adjust PD prescription.
Pediatric patients [28]	Preferred for home-based therapy, flexibility, and psychosocial development.	Transition to APD may improve adherence in adolescents.
Preference for home-based therapy/autonomy	Improves quality of life and reduces dependency on center-based schedules.	Ensure caregiver training before initiating home PD.
Active candidate for kidney transplant	KT offers superior survival and quality of life. PD may better preserve vascular access sites and RRF, facilitating transplant readiness.	Strong preference for PD as a bridge to transplant.
*Factors favoring HD*		
Recurrent PD-related infections (>2 episodes in 6 months)	High peritonitis risk may warrant transition to HD.	Switch to HD if peritonitis recurs despite antibiotic prophylaxis.
Peritoneal membrane dysfunction (high transporter status)	Compromised ultrafiltration and solute clearance efficiency.	Assess membrane status via peritoneal equilibration test.
High catabolism (e.g., severe infection, burns)	Provides more efficient and rapid solute clearance.	Use high-flux HD for patients with blood urea nitrogen >100 mg/dL.
High/uncontrolled immunologic activity (e.g., ongoing serologic activity, recent major flare)	Requires more aggressive immunosuppression, increasing infection risk.	The controlled environment of HD units allows for close monitoring.
Immediate need for RRT (e.g., life-threatening hyperkalemia, pulmonary edema)	HD enables rapid correction of metabolic and fluid disturbances.	Favors HD for initial emergency treatment. Can transition to PD once stabilized.
Unsuitable for self-care (e.g., cognitive/visual impairment, lack of caregiver)	In-center HD provides necessary supervision and support.	Consider assisted PD only if caregiver support is available.

PD: peritoneal dialysis; HD: hemodialysis; LN: lupus nephritis; ESRD: end-stage renal disease; APD: automated peritoneal dialysis; RRF: residual renal function.


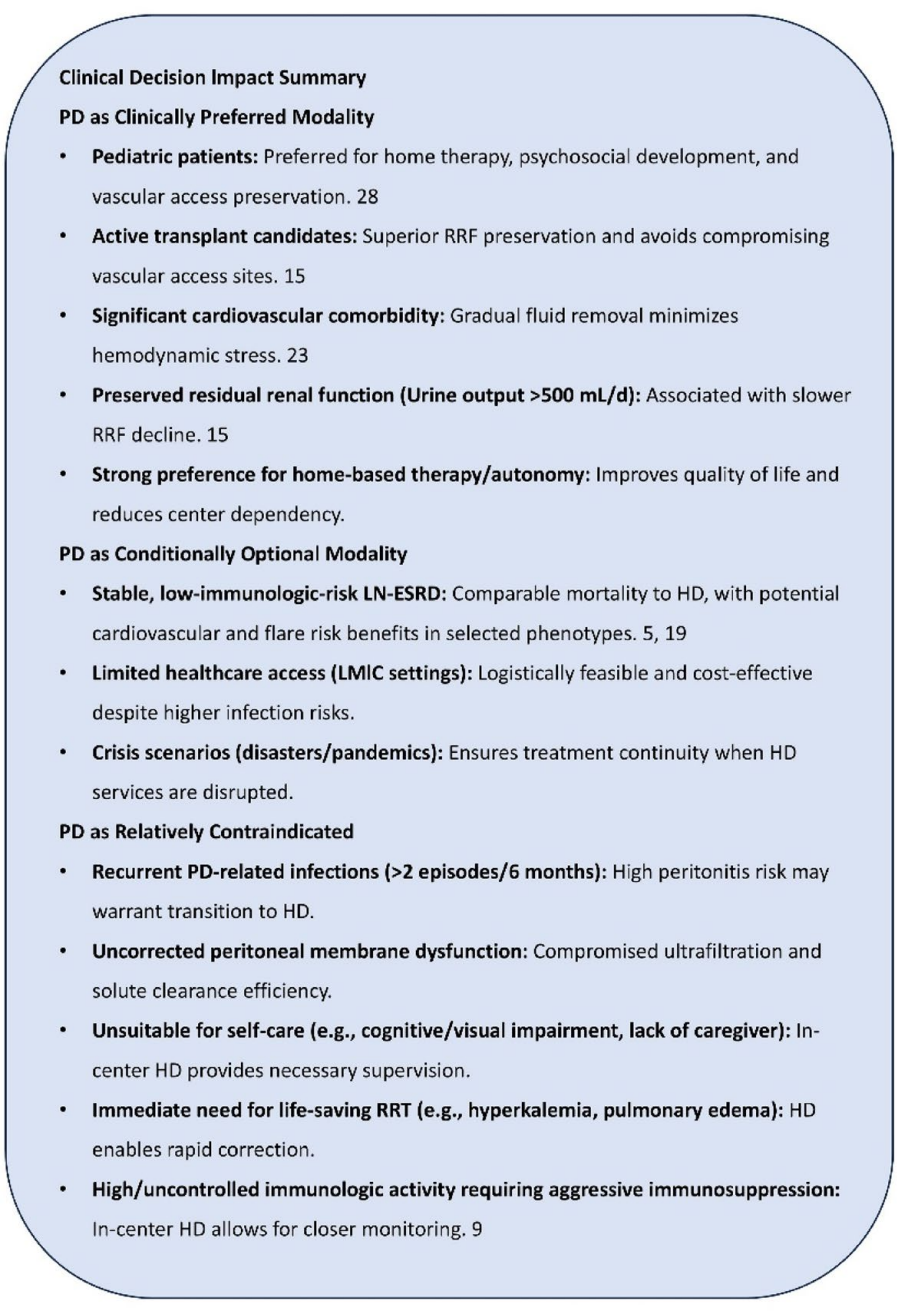


## Immunosuppressant management in PD patients

While dialysis prolongs survival, it does not address the underlying pathogenesis of SLE. Unlike extracorporeal therapies such as plasmapheresis, PD does not meaningfully remove circulating immune complexes. Although dialysis may help reduce SLE activity through potential clearance of inflammatory mediators, it does not completely prevent disease flare-ups. Therefore, ongoing monitoring of disease activity in SLE patients is crucial, even after the initiation of dialysis. Appropriate doses of steroids and hydroxychloroquine are necessary [32,33]. An SLE flare can be difficult to distinguish from other conditions, including infection, insufficient dialysis, and the effects of ESRD itself. Kim et al. [22] demonstrated that over 25% of patients with SLE experienced disease flares while on dialysis, with hematologic symptoms, especially thrombocytopenia, being the most frequent. Continued monitoring and suitable interventions, including immunosuppressive treatment, should be considered for SLE patients receiving dialysis. A total of 13 PD patients were administered intravenous methylprednisolone, oral prednisone, and mycophenolate, resulting in a significant reduction in serum creatinine levels. During the follow-up period, 10 patients discontinued PD, while three patients continued PD until the end of the follow-up.

For inadequate renal response and refractory LN, ACR 2024 recommends triple therapy: glucocorticoids + (mycophenolic acid analogs + belimumab, mycophenolic acid analogs + calcineurin inhibitor, or cyclophosphamide + belimumab) [34]. There is a lack of consensus regarding the ideal immunosuppressive regimens for LN patients undergoing PD therapy. Generally, patients with inactive LN are maintained on a low dose of steroids, optionally in combination with cytotoxic drugs like azathioprine or cyclosporine in maintenance PD patients [10]. Binda et al. [35] reported that an LN patient who began belimumab treatment while on PD experienced resolution of arthralgia, improvement in immunological parameters, and a reduction in prednisone dosage within a few months. Another study [36] also showed that belimumab is effectively and safely applied in LN patients undergoing PD. For PD patients with active LN, aggressive treatment with steroids and immunosuppressants is required. Dialysis combined with sufficient immunosuppressive medication can lead to significant improvement in 10–28% of patients with LN-ESRD, to the extent that they no longer require further dialysis [5].

The treatment landscape for LN has evolved significantly with the approval of several targeted immunotherapies, including voclosporin (calcineurin inhibitor), anifrolumab (anti-type I interferon receptor), and deucravacitinib (TYK2 inhibitor). Their use in LN patients requiring PD warrants special consideration due to the unique interplay between dialysis, systemic immunity, and drug pharmacokinetics. For voclosporin, therapeutic drug monitoring is crucial as PD may not significantly clear this predominantly hepatically metabolized drug, potentially increasing the risk of calcineurin inhibitor-related nephrotoxicity even in anuric patients. Anifrolumab’s large molecular size suggests minimal peritoneal clearance, making it suitable for PD patients. In terms of reducing infection risk, anifrolumab showed the most potential for superior efficacy over rituximab and belimumab [37]. The oral agent deucravacitinib presents a convenient option, but its pharmacokinetics in ESRD remain largely unstudied; risks of upper respiratory tract infection and nasopharyngitis necessitate careful benefit-risk assessment [38].

In addition to managing fluid balance and uremic toxins, PD provides the physiological stability required for immunosuppressive regimens. Immunosuppressants, while beneficial, can be a double-edged sword due to their side effects, which are closely tied to their therapeutic benefits. Thus, enhancing patient education is vital to ensure they are informed about their medication dosages and potential side effects, such as amenorrhea, menstrual irregularities, and leukopenia, with the aim of improving treatment adherence. Conducting regular follow-ups to monitor medication compliance is crucial for promoting adherence to prescribed treatments. It is also important to communicate the dangers of abruptly discontinuing medication to patients and to adhere strictly to medical advice on gradually decreasing dosages, expressly forbidding any unauthorized reduction or cessation of medication to prevent disease relapse from rapid steroid tapering.

## Prescription of PD in LN

We propose the ‘PD immune–microenvironment interaction hypothesis’ to explain how PD may modulate inflammatory dynamics and flare risk in LN beyond solute clearance. This hypothesis posits that PD systemically influences lupus activity through three interconnected mechanisms: first, continuous clearance of middle-molecular-weight inflammatory mediators such as interleukin-6 (IL-6) and complement components), poorly removed by HD, may reduce systemic inflammatory burden. Second, repetitive mild immunogenic stimulation of the peritoneum may induce ‘immunological conditioning,’ promoting regulatory immune responses. Third, preservation of RRF maintains endogenous clearance of autoimmune complexes, preventing systemic accumulation. This framework may explain the reduced flare rates observed in some PD studies, as continuous PD may foster a more stable immunologic milieu than the intermittent inflammatory spikes associated with HD. It also suggests that biocompatible dialysis solutions could offer added immunomodulatory benefits. While this hypothesis requires validation through biomarker and immune profiling studies, it offers a mechanistic basis for understanding how dialysis modality may influence lupus activity.

Although PD is an effective treatment option for LN patients, clinical challenges persist, such as infections related to the procedure, outcome issues with peritoneal tolerance, and PD prescription, among others. PD prescription is crucial for refining treatment strategies and optimizing the use of PD in LN. The ISPD (International Society for Peritoneal Dialysis) guidelines recommended prophylactic antibiotics before PD catheter insertion to prevent peritonitis, as well as intraperitoneal antibiotic prophylaxis prior to colonoscopy or invasive gynecological procedures in PD patients. In cases where peritonitis occurs, empiric intraperitoneal antibiotic therapy should be initiated as soon as possible after obtaining microbiological specimens, with antibiotic coverage targeting both Gram-positive and Gram-negative bacteria [39]. Proença de Moraes et al. [40] analyzed SLE and clinical outcomes in PD patients, whose PD modalities included CAPD and automated peritoneal dialysis (APD). They found that APD, when used as the initial PD modality, was an independent predictor of technique failure.

Zhou et al. [12] analyzed the PD treatment regimen for patients with severe LN complicated by significant organ dysfunction. After the insertion of the PD catheter, patients underwent three to four daytime exchanges with 1 L of 1.5% dextrose solution. After one week, the PD prescription was gradually adjusted to 2 L of dextrose solution (1.5% or 2.5%, depending on RRF and ultrafiltration), with three or four daytime exchanges, including DAPD and CAPD. The patients showed significant improvements in serum creatinine, albumin, and hemoglobin levels. Furthermore, 10 out of 13 patients experienced recovery of renal function and had their PD catheters removed.

Personalized dialysate selection is crucial for optimizing long-term outcomes in LN patients. For flare-prone patients with active serology or a history of recurrent lupus activity, minimizing systemic glucose absorption through the use of low-glucose degradation product (GDP) solutions or icodextrin may be beneficial. This approach reduces the metabolic and inflammatory burden associated with conventional glucose-based solutions, potentially mitigating one trigger of immune dysregulation. For volume-sensitive LN patients, particularly those with concomitant heart failure or hypertension, icodextrin provides sustained ultrafiltration without the glucose load, enabling more effective fluid management and reducing hemodynamic stress. The integration of peritoneal equilibration test (PET) results further refines this strategy: high transporters may benefit from APD with icodextrin for long dwells, while low transporters can often be managed with CAPD using biocompatible glucose solutions. This tailored approach to dialysate optimization – matching solution characteristics to individual patient’s clinical phenotype, membrane transport status, and lupus activity – represents a key component of precision dialysis in LN-PD care.

## Comparative PD management across LN trajectories

The application and optimization of PD vary significantly depending on whether LN patients present with AKI, CKD, or have progressed to ESRD. Each scenario entails distinct therapeutic goals, prescription strategies, approaches to integrating immunosuppression, and expected outcomes. Table 3 provides a comparative overview of PD management across these three clinical trajectories, synthesizing key considerations to guide personalized treatment planning and future research directions.

**Table 3.** PD in LN: AKI vs. CKD vs. ESRD – goals, prescription strategy, immunosuppression integration, and outcomes.

Aspect	LN with AKI	LN with CKD	LN with ESRD
Primary goal	Bridge to renal recovery; support ongoing immunosuppression.	Delay CKD progression; preserve RRF; manage complications.	Long-term RRT; maintain quality of life; bridge to transplant.
PD prescription strategy	Initial low-volume, frequent exchanges (e.g., 1–1.5 L, 4–5 cycles/day); gradual increase as tolerated.	Titrated to RRF and urine output; often CAPD or flexible APD; focus on steady-state metabolic control.	Full-dose, RRF-independent regimen; often APD for lifestyle flexibility; strict volume management.
Immunosuppression integration	Aggressive, full-dose therapy supported by PD’s hemodynamic stability; monitor for infection.	Reduced but active maintenance therapy; balance between lupus control and infection risk.	Minimal or lupus-specific (e.g., HCQ) only; focus on preventing flares with low immunosuppression burden.
Expected outcomes and future directions	High potential for renal recovery and PD discontinuation; biomarker-guided weaning.	Slower RRF decline; reduced cardiovascular stress; home-based therapy benefits.	Long-term technique survival; peritonitis prevention; transition planning to transplant or HD.

PD: peritoneal dialysis; AKI: acute kidney injury; CKD: chronic kidney disease; RRF: residual renal function; HD: hemodialysis; LN: lupus nephritis; ESRD: end-stage renal disease; CAPD: continuous ambulatory peritoneal dialysis; APD: automated peritoneal dialysis.

## Prognosis and long-term outcomes

**Figure 2.**
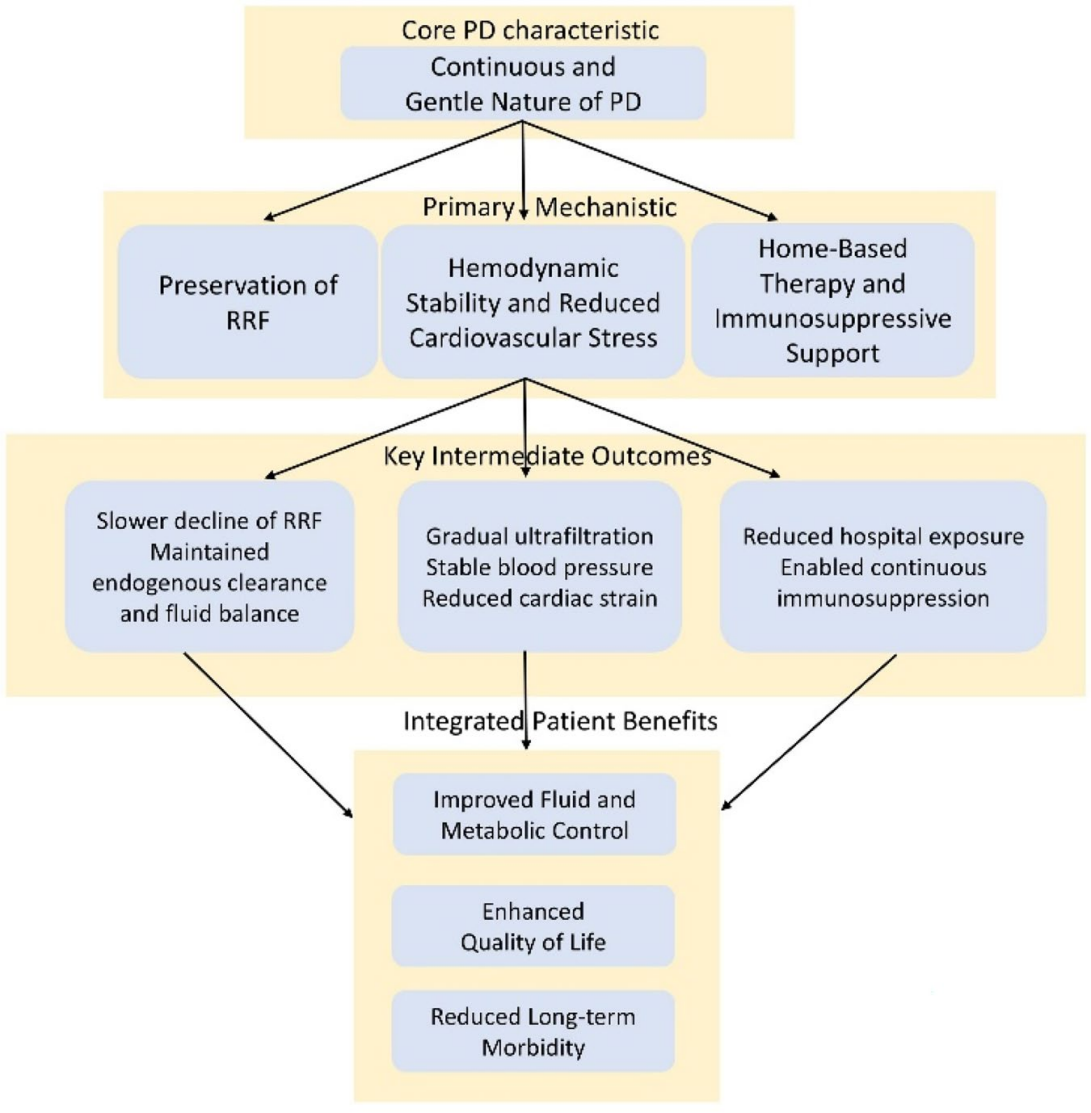
Mechanistic pathways linking PD to improved renal, hemodynamic, and immunologic outcomes in LN. Schematic illustration of the proposed mechanistic pathways through which the continuous and gentle nature of PD translates into integrated clinical benefits for patients with lupus nephritis. The diagram delineates the causal cascade from the core characteristic of PD (left) through primary mechanistic drivers (center-left), leading to key intermediate outcomes (center-right), and culminating in ultimate patient benefits (right). The three interconnected vertical pathways highlight: (1) preservation of RRF: the gentle therapy slows RRF decline, maintaining endogenous clearance. (2) *Hemodynamic stability*: Gradual ultrafiltration promotes stable blood pressure and reduces cardiac strain. (3) *Home-based care & immunosuppressive support*: Reduced hospital exposure enables consistent immunosuppressive management. These pathways collectively contribute to improved fluid/metabolic control, enhanced quality of life, and reduced long-term morbidity. PD: peritoneal dialysis; RRF: residual renal function.

Based on a large prospective cohort study [40], PD therapy was found to be a safe and effective treatment for SLE patients requiring PD, with outcomes for mortality, technique failure, and time to the first peritonitis episode comparable to those of non-SLE patients. Findings from the ERA Registry [3] showed that the mortality rate between SLE and non-SLE patients did not show a significant difference on both day 1 and day 91 after initiating PD treatment. Zhou et al. [12] demonstrated that PD had low infection and mortality rates, along with a high rate of renal function recovery, suggesting that PD may be a viable treatment option for patients with severe LN and AKI who require ongoing immunosuppressive therapy. PD is also an appropriate therapy for patients experiencing high catabolism, oliguria, anuria, severe malnutrition, water-sodium retention, prerenal failure, and cardiovascular issues. However, the careful selection of appropriate immunosuppressive therapy during PD is essential for optimal outcomes. The clinical benefits observed in LN patients treated with PD, as compared to HD, can be attributed to its distinct mechanistic pathways. As summarized in Figure 2, PD exerts its positive effects through three interconnected core mechanisms: the preservation of RRF, the maintenance of hemodynamic stability, and unique immunomodulatory advantages.

However, some studies have found that LN patients have poor outcomes after treatment with PD. A matched case-control study [41] found that LN patients on PD had lower survival rates, along with higher rates of infection and hospitalization in long-term clinical outcomes. Another study [9] similarly reported an increased risk of infection associated with PD, suggesting that SLE itself may impair the immune function of patients with renal failure. Furthermore, long-term use of glucocorticoids and immunosuppressants is one of the core reasons for the increased risk of infection in LN-PD patients. Sule et al. [42] found that infections were responsible for 20% of deaths in adult patients with ESRD secondary to SLE. However, Sabucedo and Contreras [43] reported a decline in infection-related mortality rates. In a study by Contreras et al. [25], it was shown that mortality from CVD was more prevalent than mortality from infection-related causes. Chang et al. [26] presented contrasting findings, highlighting infection-related mortality as the primary cause of death among ESRD patients with SLE. Notably, their cohort study was limited to Asian patients, who may exhibit unique exposures and susceptibilities to risk factors associated with infectious events. Broder et al. [44] observed that SLE patients with ESRD who had positive antiphospholipid antibodies, particularly antiphospholipid/lupus anticoagulant, had a higher risk of all-cause mortality compared to similar patients who did not have these antibodies in SLE-ESRD.

Although PD can serve as a bridge to transplantation in LN-ESRD, current studies and guidelines consistently recommend KT as the definitive treatment to ultimately improve patient outcomes. Brilland et al. [45] showed for patients with LN-ESKD, KT confers a significant survival advantage over remaining on dialysis; the early evaluation of transplant eligibility and timely referral to transplant centers are of paramount importance to optimize outcomes. The 2024 Kidney Disease: Improving Global Outcomes (KDIGO) LN guidelines recommend patients with LN who progress to kidney failure may be treated with HD, PD, or KT, and KT is preferred over long-term dialysis [46]. The 2024 American College of Rheumatology (ACR) LN guidelines recommend KT over dialysis for patients with LN-ESRD; transplantation can proceed without requiring complete clinical or serologic remission, provided there is no major extrarenal organ involvement, with ongoing rheumatology follow-up post-transplant [34]. These guidelines do not explicitly recommend whether PD or HD is more suitable for LN-ESRD patients. They only indicate that PD carries a higher risk of peritonitis, while HD is more frequently associated with bloodstream infections and thrombosis related to vascular access. In summary, most large registry studies suggest comparable mortality between PD and HD in LN-ESRD patients, but infection risk remains a significant concern in those undergoing PD, particularly under high-dose steroid therapy. Once PD-associated peritonitis occurs, active therapeutic intervention should be initiated in accordance with the ISPD 2022 Peritonitis Guidelines [39].

The outcomes of PD in LN vary significantly across different resource settings. In high-income countries, robust infrastructure and comprehensive support systems typically result in lower peritonitis rates and higher technique survival. In contrast, low- and middle-income countries (LMICs) often struggle with limited access to biocompatible solutions, higher baseline infection rates, and a shortage of specialized care, increasing the risk of PD-related complications. Nevertheless, PD remains a critically feasible option in LMICs due to its lower initial costs and reduced reliance on centralized facilities compared to HD. Moreover, PD demonstrates exceptional resilience during disasters – such as pandemics, natural catastrophes, or conflict – enabling continuity of care when HD services are disrupted. Strengthening supply chains, simplifying training, and integrating PD into crisis-response plans are essential strategies to enhance its accessibility and safety in resource-limited settings.

## A proposed PD-based lupus recovery index

The heterogeneous clinical course of LN patients on PD necessitates a standardized tool for prognostication and personalized management. In response to this need, we propose a novel conceptual framework: the PD-based lupus recovery index (PD-RI). This multidimensional scoring system is designed to quantify the likelihood of renal recovery and stratify the risk of lupus flare in LN patients initiating PD, integrating four critical, readily assessable clinical domains, as detailed in Table 4.

**Table 4.** The PD-based lupus recovery index (PD-RI): a conceptual scoring framework.

Domain	Score = 1	Score = 2	Score = 3
Residual renal function	Urine output <200 mL/day; Urea clearance <2 mL/min	Urine output 200–500 mL/day; Urea clearance 2–4 mL/min	Urine output >500 mL/day; Urea clearance >4 mL/min
Systemic lupus activity	Active serology (↑ anti-dsDNA, ↓ C3/C4); Major extra-renal manifestations	Stable but abnormal serology; Minor non-renal symptoms	Normalized serology; No clinical extra-renal activity
PD efficacy and tolerance	Inadequate solute clearance (*Kt*/*V* < 1.7); Fluid overload Frequent membrane-related issues	Adequate clearance (*Kt*/*V* 1.7–2.0); Generally stable volume	Optimal clearance (*Kt*/*V* > 2.0); excellent volume control; No membrane dysfunction
Immunosuppressive responsiveness	Refractory to standard therapy; Frequent flares on therapy	Controlled with full-dose therapy	Controlled on low-dose/maintenance therapy

PD: peritoneal dialysis; *Kt*/*V*: urea clearance index.

The PD-RI evaluates: (1) RRF: a cornerstone of recovery potential and overall homeostasis; (2) systemic lupus activity: reflecting the underlying autoimmune drive. (3) *PD efficacy and tolerance*: Ensuring adequacy of dialysis and membrane health. (4) *Immunosuppressive responsiveness*: Indicating the disease’s susceptibility to control. Each domain is assigned a score from 1 (least favorable) to 3 (most favorable). The sum of these scores (range: 4–12) places the patient in one of three prognostic categories: high PD-RI (10–12): high probability of renal recovery/low flare risk. Characterized by significant RRF, quiescent lupus, effective PD, and good immunosuppressive response. Management should focus on RRF preservation and cautious immunosuppression reduction. *Focus*: RRF preservation, aggressive IS weaning; moderate PD-RI (7–9): guarded recovery potential/moderate flare risk. Represents an intermediate state. Management requires close monitoring and balanced optimization of all parameters. *Focus*: Close monitoring, optimize all parameters; low PD-RI (4–6): low probability of renal recovery/high flare risk. Indicates advanced disease with poor RRF, active lupus, and suboptimal treatment response. The focus shifts to long-term dialysis management, rigorous flare prevention, and preparation for transplantation. *Focus*: Long-term dialysis management, rigorous flare prevention, and transplant planning.

The PD-RI provides a structured framework to move beyond subjective assessment, enabling more standardized prognostication, tailored treatment intensity, and strategic planning for LN patients on PD. Future prospective studies are warranted to validate and refine this index.

## Emerging technologies and future

The future of PD in LN lies at the confluence of several emerging technologies, paving the way for an era of precision dialysis and digital PD. This includes the development of integrating these technologies: next-generation biocompatible dialysates, digital health, remote patient monitoring (RPM), multi-omics biomarker discovery, and novel immunomodulatory agent.

The development of next-generation biocompatible dialysates is fundamental. Neutral-pH, low-GDP solutions and those with alternative osmotic agents (e.g., icodextrin, l-carnitine, and xylitol [47]) better preserve RRF and peritoneal membrane integrity. Their adoption is particularly relevant for LN patients, who may be more susceptible to membrane damage from systemic inflammation and conventional solutions. Furthermore, digital health and RPM platforms are revolutionizing home-based care [32]. For infection-prone LN patients, these systems enable early peritonitis detection through automated effluent analysis and facilitate tighter management of fluid balance and blood pressure, addressing critical comorbidities. The application of multi-omics and biomarker discovery promises a shift toward personalized therapy. Proteomic and metabolomic profiling of serum and peritoneal effluent could yield signatures predictive of peritonitis risk or disease flare, allowing for preemptive treatment adjustments. Finally, the convergence of PD with novel immunomodulatory agents opens new avenues. Research into the safety and efficacy of targeted biologics in the PD population is needed, exploring their potential to concurrently control systemic lupus activity and modulate the local peritoneal immune environment.

Integrating these technological advancements with the principles of precision dialysis is pivotal for enhancing the safety, efficacy, and long-term sustainability of PD therapy for LN patients. The integration of artificial intelligence (AI) and digital health is transforming PD management in LN toward proactive and personalized care. RPM platforms enable at-home tracking of vitals, fluid status, and treatment adherence, permitting early intervention and fewer clinic visits – a key benefit for immunocompromised LN patients. Emerging wearable sensors enable early detection of peritonitis by analyzing effluent dialysate for signs of infection, such as cloudiness or elevated white blood cell counts, allowing for pre-symptomatic intervention. Tele-PD platforms enhance virtual clinical support, improving patient education and treatment adherence. The concept of ‘digital twins’ – virtual models of a patient’s peritoneal membrane – simulates responses to different PD regimens, supporting optimized prescription design. Additionally, machine learning-driven predictive analytics applied to electronic health records and real-world PD data can stratify patients by risks of technique failure, hospitalization, or lupus flare, facilitating preemptive management. Together, these technologies advance high-precision home-based dialysis care, offering LN patients closer monitoring while reducing infection exposure and improving quality of life.

To bridge the gap between current evidence and future clinical practice, we propose a Translational Research Gaps and Trial Roadmap (Table 5), outlining priority areas for investigation in LN patients undergoing PD.

**Table 5.** Translational research gaps and trial roadmap for PD in LN.

Priority research area	Core research gap	Suggested study design	Primary outcomes	Rationale (with citations)
PD immunomodulatory mechanisms	Validation of the ‘PD immune–microenvironment interaction hypothesis’ (proposed in this review)	Prospective observational cohort with multi-omics profiling (serum/peritoneal effluent)	Differences in cytokine (IL-6, TNF-α) clearance, regulatory T-cell subsets, and flare rates between PD/HD	Current evidence suggests PD may reduce inflammatory burden, but mechanistic data on cytokine clearance and immune conditioning remain scarce [5,22]. This review would elaborate the hypothesis and explain PD’s potential flare-lowering effects.
PD + targeted biologics efficacy	Safety/efficacy of belimumab/other biologics in LN-PD patients (no consensus on regimens [35])	Multicenter, open-label phase 2 trial	Flare-free survival, peritonitis incidence, and steroid-sparing effect	Binda et al. [35] and Liu et al. [36] reported preliminary efficacy of belimumab in LN-PD patients, but large-scale trials are lacking to confirm long-term safety and optimal dosing.
Cytokine-load clearance comparison	Superiority of PD vs. HD in removing lupus-related inflammatory mediators (unproven [5])	Prospective cross-over study (PD → HD or vice versa)	Middle-molecular-weight cytokine levels (IL-6, complement C3a) and systemic inflammation scores	Gong et al. [5] noted PD’s potential cardiovascular benefit but did not measure inflammatory mediator clearance – this study would directly test whether PD’s continuous clearance targets lupus-specific cytokines.
AI-driven peritonitis prediction	Early detection of peritonitis in high-risk LN-PD patients (current monitoring has low sensitivity [39])	Retrospective cohort with AI/ML modeling (using effluent cell count, clinical data, and RPM metrics)	Sensitivity/specificity of AI model for peritonitis prediction (vs. standard monitoring)	ISPD guidelines [39] emphasize the need for improved peritonitis detection in immunosuppressed patients; AI tools could address this gap by integrating real-time data.
Biocompatible dialysate in LN	Impact of neutral-pH/low-GDP solutions on peritoneal membrane function (LN patients have heightened membrane vulnerability [9])	RCT comparing conventional vs. biocompatible dialysates	PET results, residual renal function decline, and membrane failure rate	Huang et al. [9] showed LN-PD patients have higher peritonitis rates and membrane stress; biocompatible solutions (e.g., icodextrin-based [47]) may mitigate this, but no RCTs have focused on LN.
Pediatric LN-PD adherence interventions	Strategies to improve adherence in adolescents transitioning to adult care (high technique failure [31])	Cluster RCT (pediatric vs. adult centers) testing APD + digital adherence tools	Technique survival, non-adherence rates, and psychosocial outcomes	Watson [31] reported 2–3× higher technique failure in adolescent LN-PD patients due to non-adherence; digital tools (e.g., RPM [14]) could improve outcomes but lack pediatric-specific testing.

PD: peritoneal dialysis; HD: hemodialysis; IL-6: interleukin-6; TNF-α: tumor necrosis factor-α; AKI: acute kidney injury; LN: lupus nephritis; ESRD: end-stage renal disease; AI: artificial intelligence; RCT: randomized controlled trial; APD: automated peritoneal dialysis; LN-PD: lupus nephritis-peritoneal dialysis; RPM: remote patient monitoring; GDP: glucose degradation product; PET: peritoneal equilibration test.

## Conclusions

The application of PD in the treatment of LN with concurrent CKD/AKI represents a valuable option in managing this complex condition. There are many challenges in clinical practice, such as the use of immunosuppressive agents, adjustments in PD prescriptions, and complications or poor prognosis associated with PD. PD is an effective RRT for LN patients, but its successful application relies on individualized prescription, complication management, and multidisciplinary collaboration. With a deeper understanding of the treatment mechanisms for LN and continuous advancements in PD technology, it is hoped that more safe and effective treatment options will be available for LN patients in the future. Ongoing innovation in PD technologies, biocompatible dialysates, and biomarker-guided immunosuppression may further improve outcomes in LN patients.

## Data Availability

The authors confirm that the data supporting the findings of this study are available within the article.
